# Microendoscope-Assisted Posterior Decompression Technique Using a 12-mm Tubular Retractor of the SYNCHA Novel Microendoscopic System for Cervical Spondylotic Radiculopathy: A Technical Note

**DOI:** 10.7759/cureus.80629

**Published:** 2025-03-15

**Authors:** Motohiro Okada, Munehito Yoshida, Kazunori Nomura, Ken-ichi Yawatari, Sae Okada

**Affiliations:** 1 Orthopaedic Surgery, Sumiya Orthopaedic Hospital, Wakayama, JPN

**Keywords:** degenerative cervical spondylosis, microendoscopic surgery, minimally-invasive spine surgery, nonmyelopathic spondylotic cervical cord compression with radiculopathy (nmsccc-r), posterior spinal decompression

## Abstract

The microendoscope-assisted posterior decompression technique for degenerative spinal disorders has gained popularity as a useful minimally invasive spinal surgery in Japan. We performed the technique in >9,000 cases of intervertebral lumbar disc herniation, lumbar spinal canal stenosis, cervical spondylotic myelopathy, and cervical spondylotic radiculopathy (CSR). We primarily used the METRx microendoscopic system (Medtronic Sofamor Danek, Minneapolis, MN) with a 16-mm tubular retractor. The SYNCHA (Teijin Nakashima Medical, Okayama, Japan) is a novel microendoscopic system developed by Yoshida. The features of this system include (1) a ball-link mechanism enabling joystick action and (2) tubular retractors ranging from 12 to 18 mm in diameter with lineup at 2 mm intervals. We are currently performing posterior decompression surgeries using a 12-mm tubular retractor that enables reduced invasiveness.

The current study describes a technique using the SYNCHA system with a 12-mm tubular retractor for CSR. The technique was employed in seven cases of CSR. Surgical procedures were performed according to the methods using a 16-mm tubular retractor as described previously by Adamson. Thanks to its joystick action, the 12-mm tubular retractor provided a consistently equivalent field of view compared to the 16-mm tubular retractor. Despite the limited working space, it was possible to perform adequate decompression by using the 12-mm tubular retractor. Microendoscope-assisted posterior decompression technique using the SYNCHA microendoscopic system with a 12-mm tubular retractor is not only safe, minimally invasive, and effective for the treatment of CSR, but it may also provide advantages over full-endoscopic spinal surgery (FESS) and biportal endoscopic spinal surgery (BESS).

## Introduction

In recent years, the number of elderly patients with degenerative cervical and lumbar disorders has been on the rise with the increase in the average life span. Conventional spinal posterior decompression techniques were associated with large skin incisions and damage to posterior supportive tissues including muscles, ligaments, facet joints, and capsules, carrying the risk of chronic neck or low back pain and spinal instability. Microendoscopic discectomy (MED) was developed as a minimally invasive surgical technique for the treatment of intervertebral lumbar disc herniation (LDH) by Smith and Foley in 1997 [1]. The microendoscope-assisted posterior decompression technique for treating degenerative spinal disorders has been gaining popularity as a useful, minimally invasive spinal surgery in Japan since Yoshida reported the first case of LDH in the country in 1998 [2]. We have treated >9,000 cases of LDH, lumbar spinal canal stenosis, cervical spondylotic myelopathy, and cervical spondylotic radiculopathy (CSR). Most patients with CSR receive conservative treatment to relieve their symptoms such as neck and upper limb pain. However, upon failure of conservative treatment or in cases of progressive or severe neurological deficit, surgical treatment is recommended.

The main advantage of the microendoscope-assisted posterior decompression technique is that the surgical field is magnified and illuminated, allowing not only minimally invasive techniques but also enabling the procedure to be performed safely and accurately. Furthermore, microendoscopic surgery has the advantage of minimizing damage to the posterior supportive tissues, which also contributes to spinal stability. We have primarily used the METRx microendoscopic system (Medtronic Sofamor Danek, Minneapolis, MN) with a 16-mm tubular retractor [2]. The SYNCHA (Teijin Nakashima Medical, Okayama, Japan) instrument is a novel microendoscopic system developed by Yoshida in 2020. The features of this system include (1) a ball-link mechanism enabling joystick action and (2) tubular retractors ranging from 12 to 18 mm in diameter with a lineup at 2 mm intervals. We are currently treating CSR cases with posterior decompression surgery using a 12-mm tubular retractor to ensure minimal invasiveness.

In this report, we describe the surgical procedure using a 12-mm tubular retractor for treating seven cases of CSR and evaluate their preliminary clinical outcomes, feasibility, and safety; we also compare it with the conventional 16-mm tubular retractor and discuss the advantages of the procedure over other microendoscopic techniques such as full-endoscopic spinal surgery (FESS) and biportal endoscopic spinal surgery (BESS).

## Technical report

**Figure 1:**
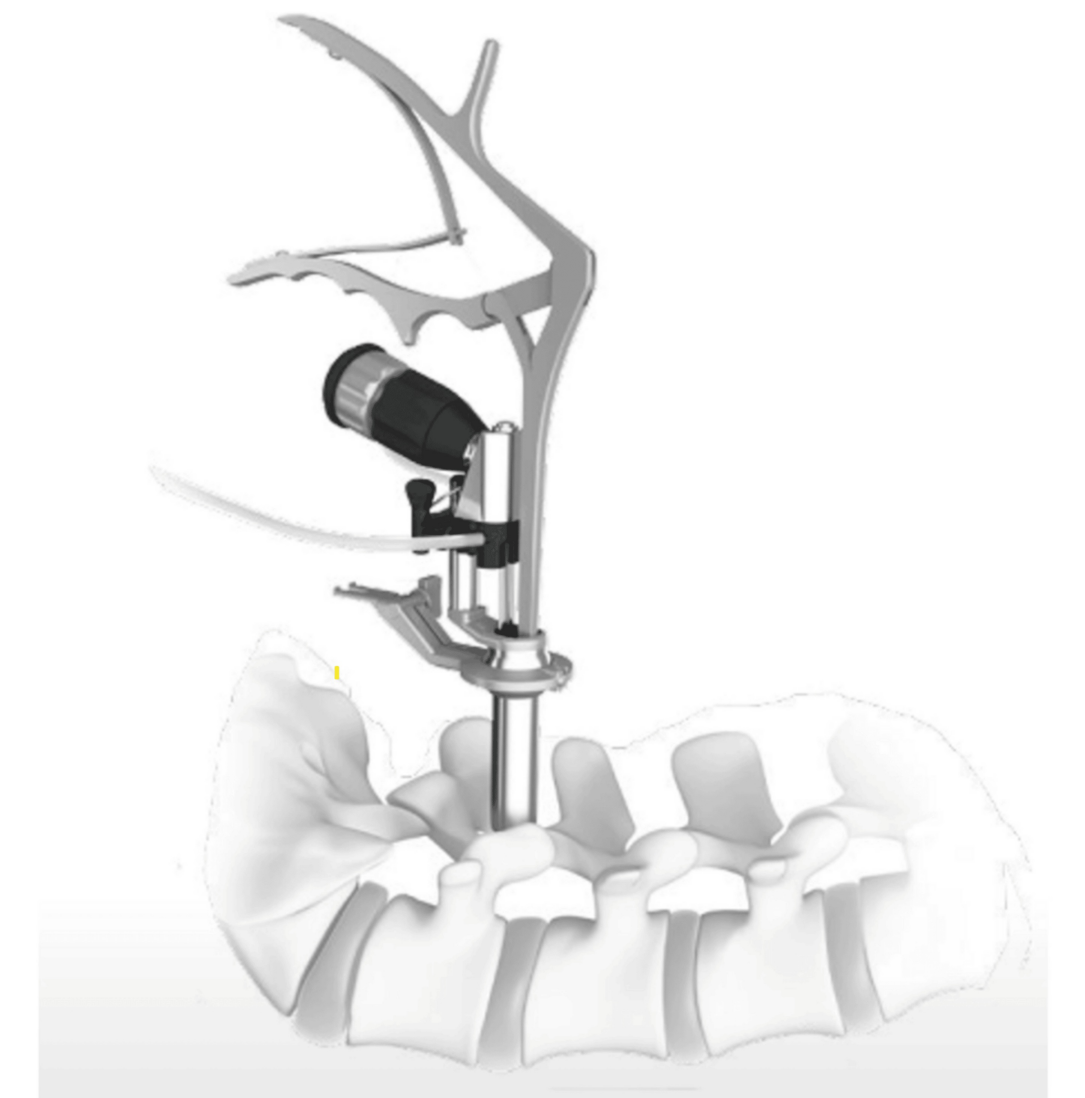
Aspects of SYNCHA (Teijin Nakashima Medical, Okayama, Japan) endoscopic system

**Figure 2:**
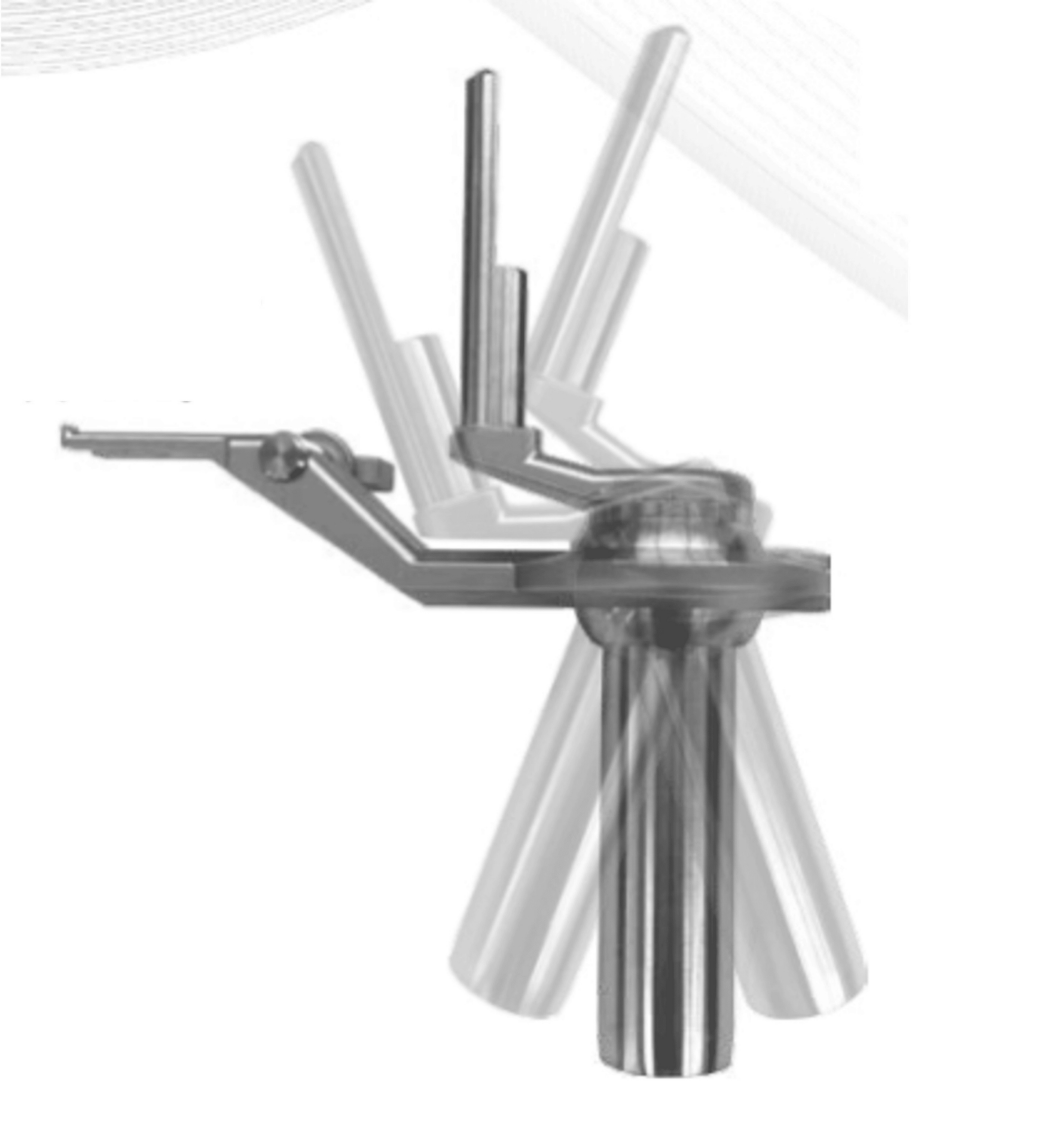
Ball-link mechanism enabling joystick action in the SYNCHA system

**Figure 3:**
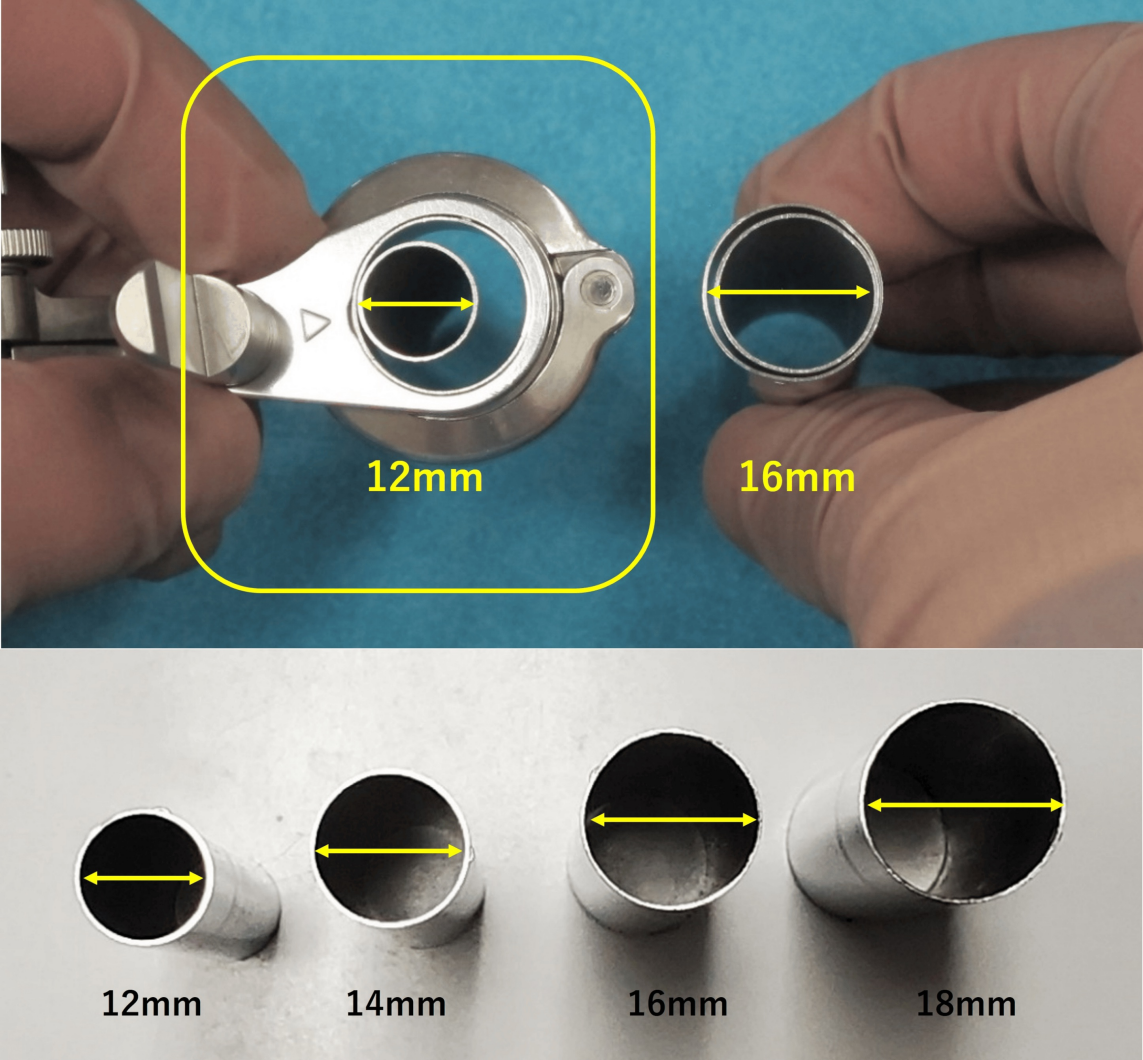
Lineup of the tubular retractors of the SYNCHA system

Figures 1-3 illustrate the SYNCHA endoscopic system and its various elements.

Surgical procedures for CSR employed a 12-mm tubular retractor and were conducted according to the methods using a 16-mm tubular retractor as described previously by Adamson [3].

**Figure 4:**
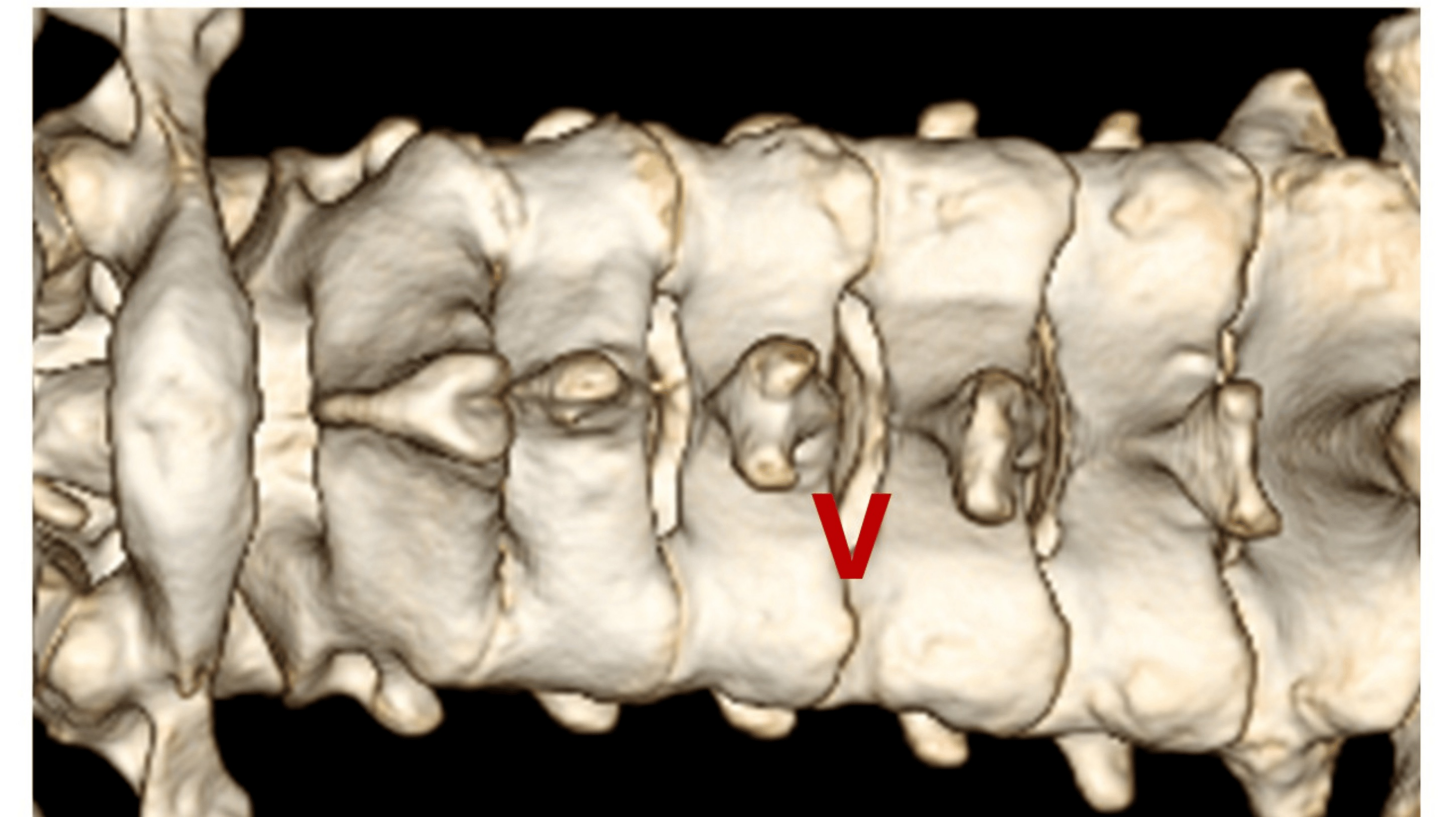
Preoperative 3D-CT image The overlap of the superior and inferior articular processes enabled the visualization of the so-called Interlaminar V CT: computed tomography

**Figure 5:**
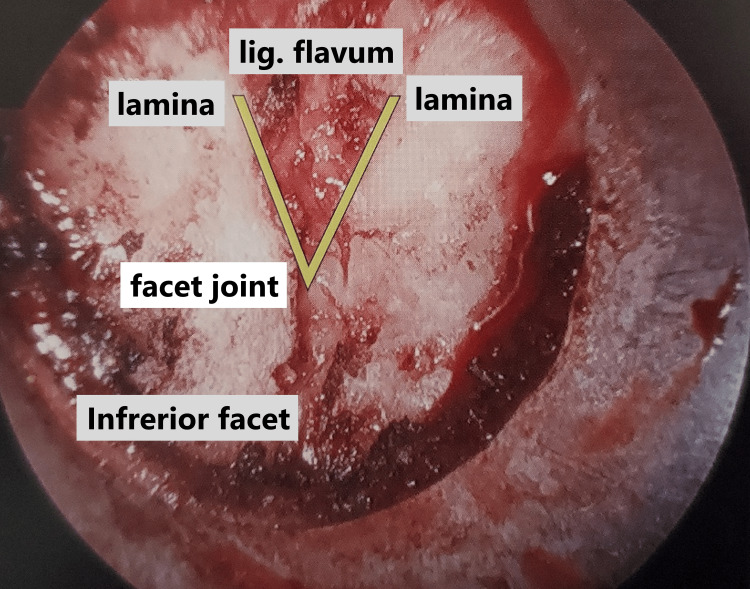
Intraoperative findings of the interlaminar V

**Figure 6:**
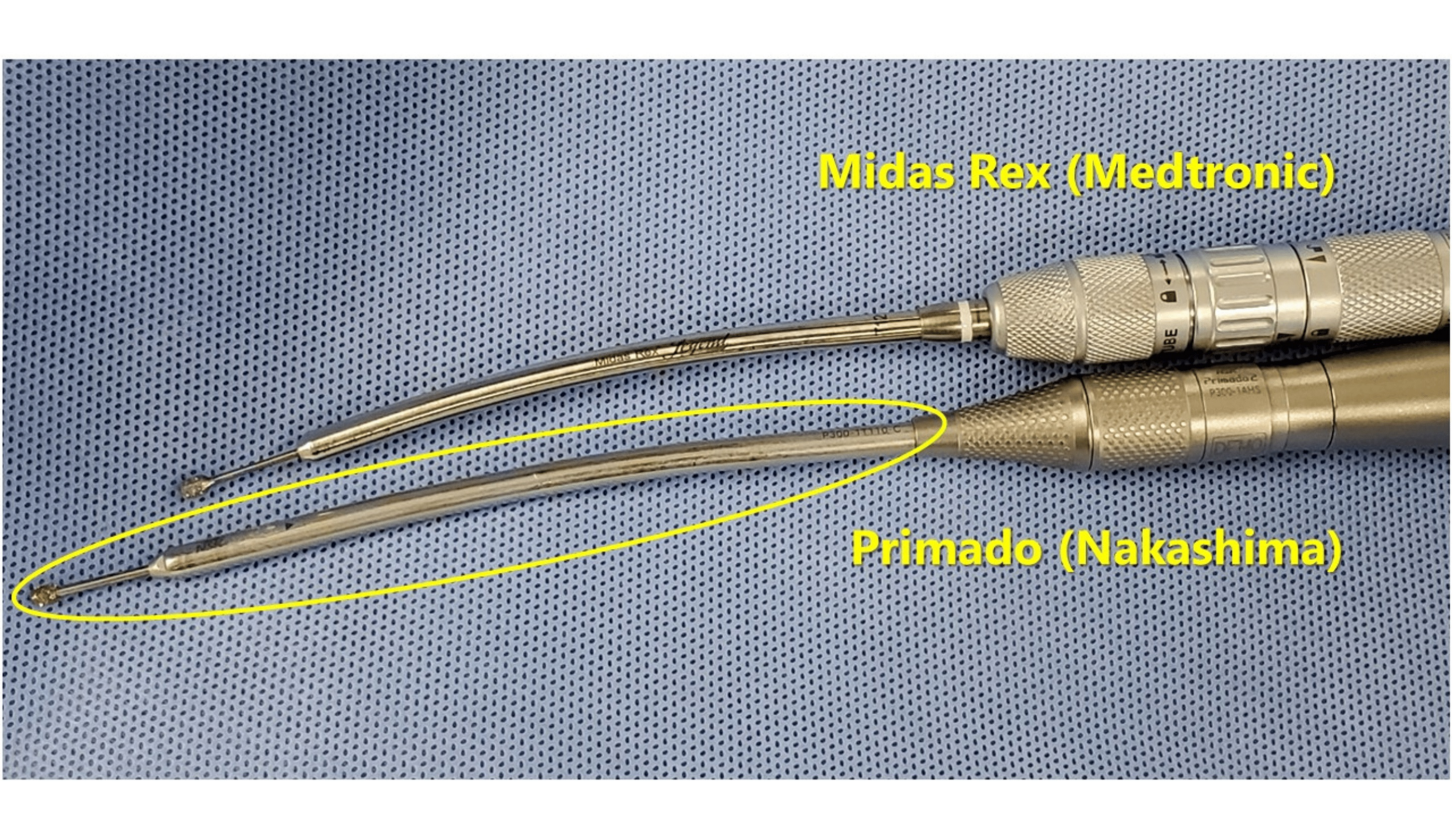
Long-curved endoscopic bar

The procedure was as follows:

The patient's head was secured in a Mayfield head holder and placed in a prone position under general endotracheal anesthesia. The neck was secured in a neutral position. The fluoroscopic C-arm was carried to the surgical site, and we marked the target level with a pen under lateral fluoroscopic guidance. The operator stood on the symptomatic side and a video monitor was located on the opposite side of the patient. An approximately 13-mm paramedian skin incision was created above the spinous process. After performing a paramedian fasciotomy on the side of the approach, the paravertebral muscles were split with serial dilators of the SYNCHA microendoscopic system. A 12-mm tubular retractor was passed over the dilators and fixed to a flexible arm mounted on the table-side rail, and an oblique-viewing microendoscope was attached to the tubular retractor. Lateral fluoroscopy confirmed the precise localization of the tubular retractor after resection of the residual musculature and soft tissues on the lamina and facet joint. The following procedures were performed microendoscopically. With the overlap of the superior and inferior articular processes, the so-called interlaminar V (Figures 4, 5) was optically visualized. A high-speed drill with a long curved endoscopic bar (Primado 2, Nakanishi, Kanuma, Tochigi, Japan; Figure 6) was used to cut the inferior articular process while performing the joystick action of the 12-mm tubular retractor repeatedly until the superior articular process was exposed.

**Figure 7:**
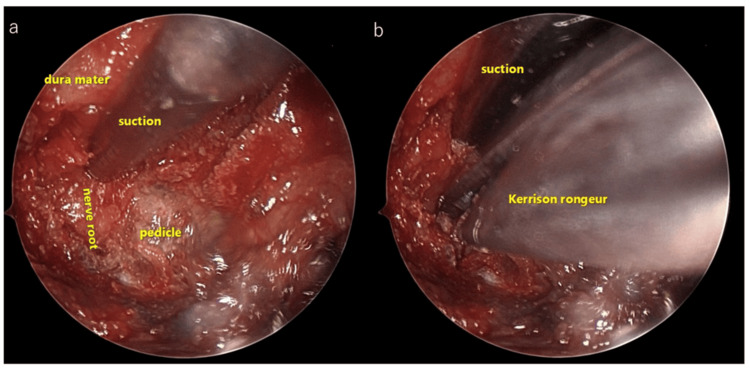
Intraoperative findings in the 12-mm tubular retractor of SYNCHA endoscopic system a: Intraoperative findings of dura mater, nerve root and pedicle in the 12-mm tubular retractor. b: The SYNCHA endoscopic system allows the use of both hands

**Figure 8:**
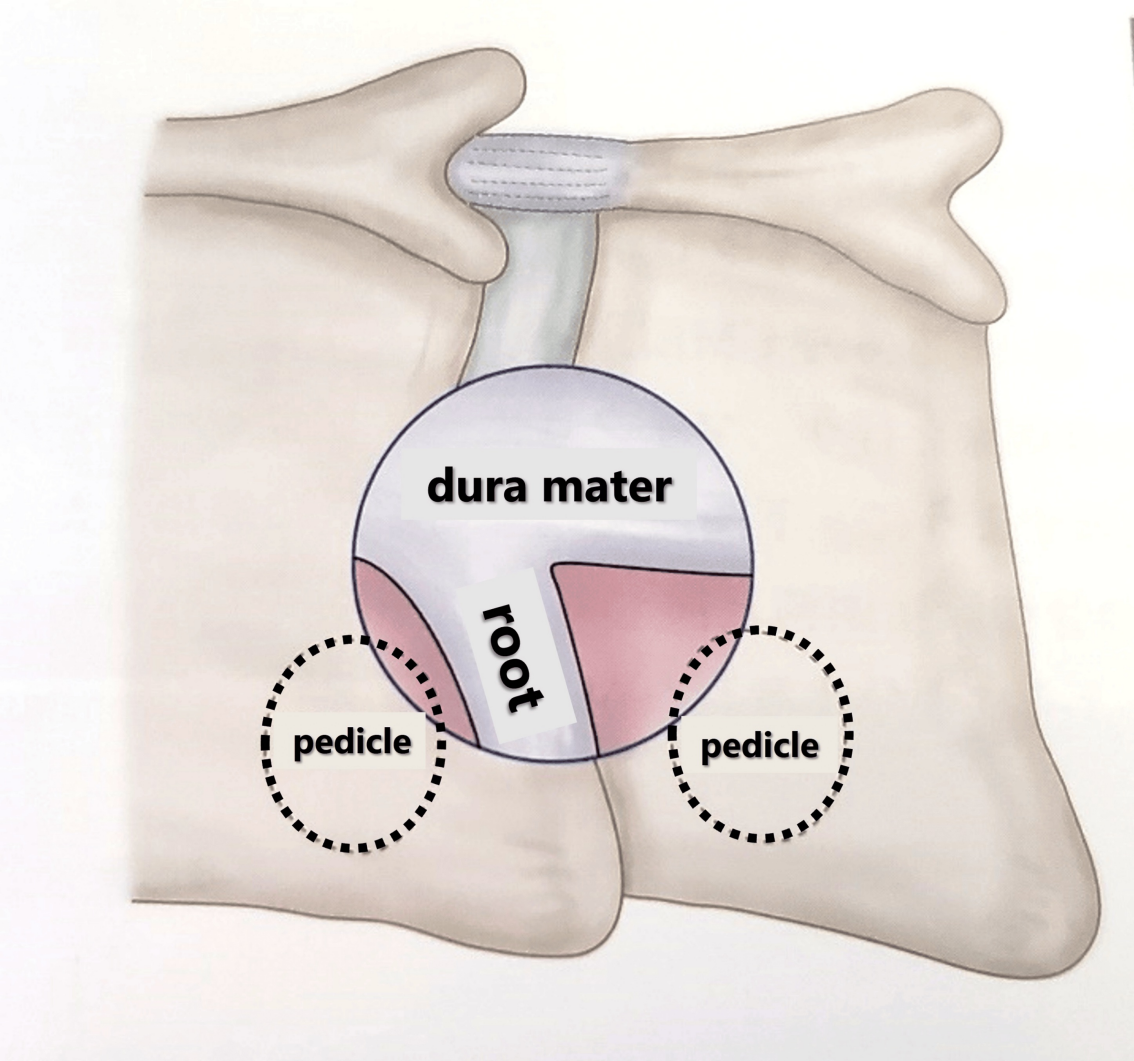
Schematic of posterior decompression for cervical spondylotic radiculopathy (CSR)

**Figure 9:**
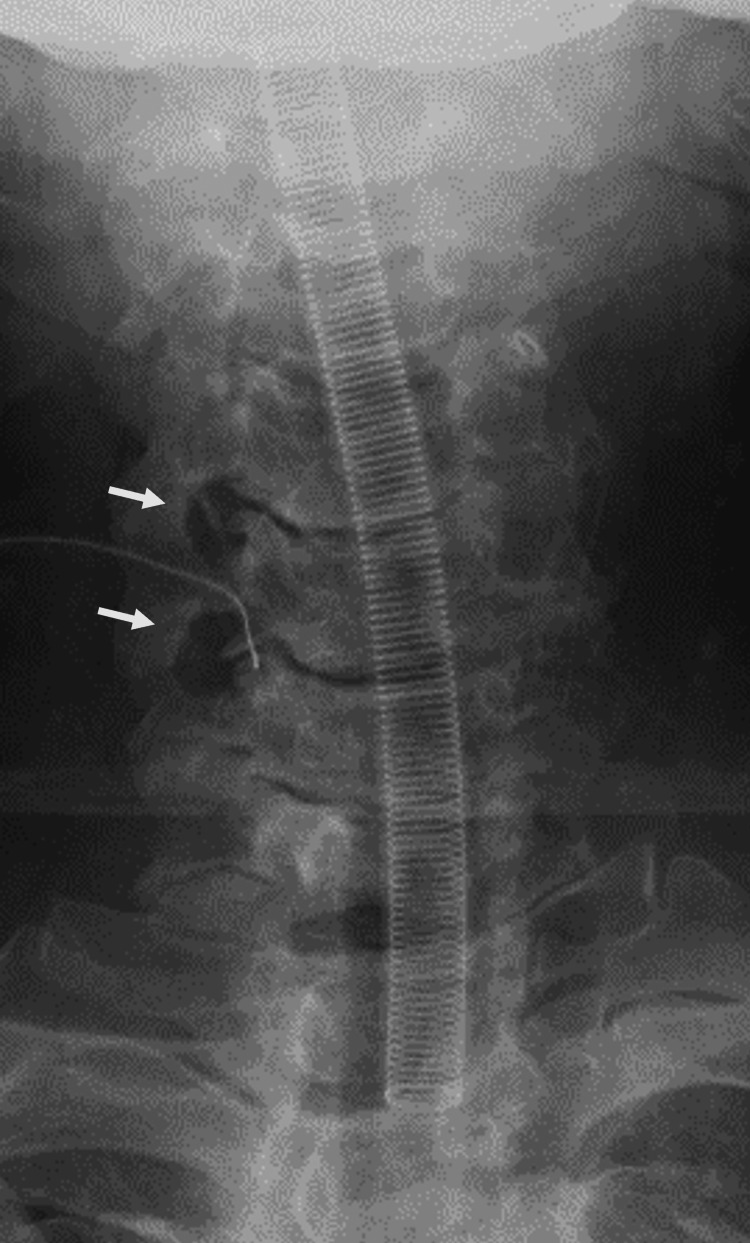
Postoperative anterior-posterior X-ray Posterior decompression is achieved beyond the Luschka joint. The arrows show the decompression sites

**Figure 10:**
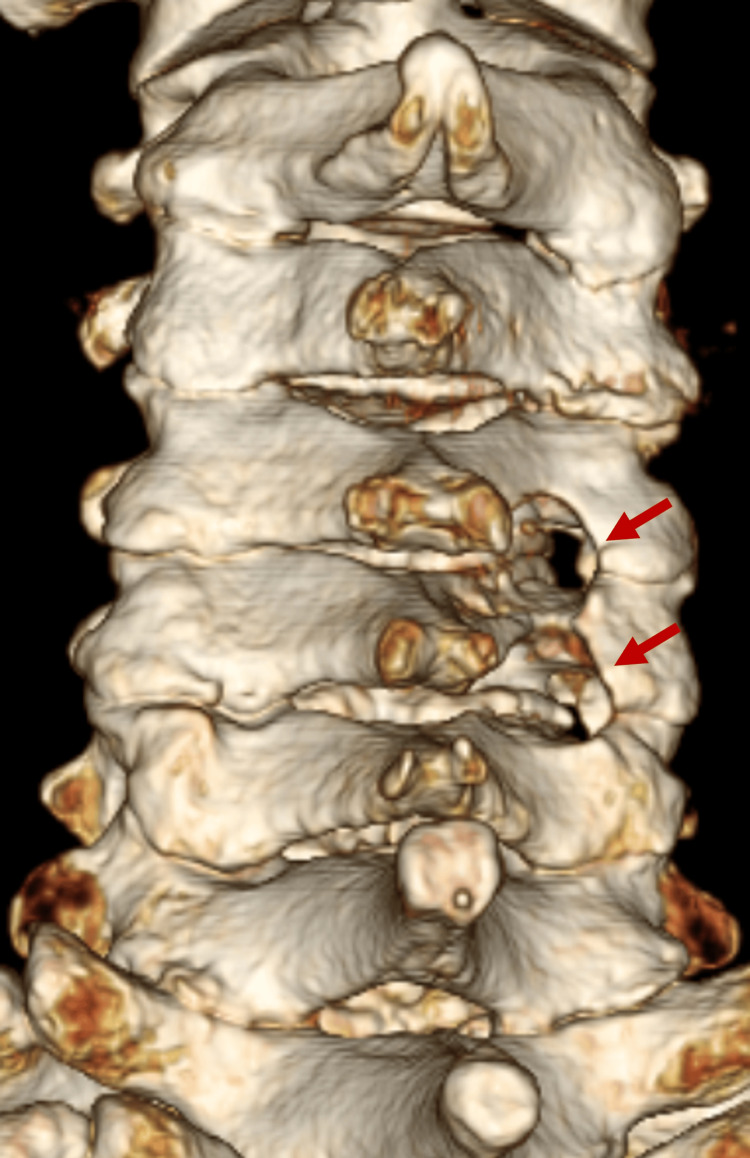
Postoperative 3D-CT image Posterior decompression is achieved beyond the Luschka joint. The arrows show the decompression sites CT: computed tomography

**Figure 11:**
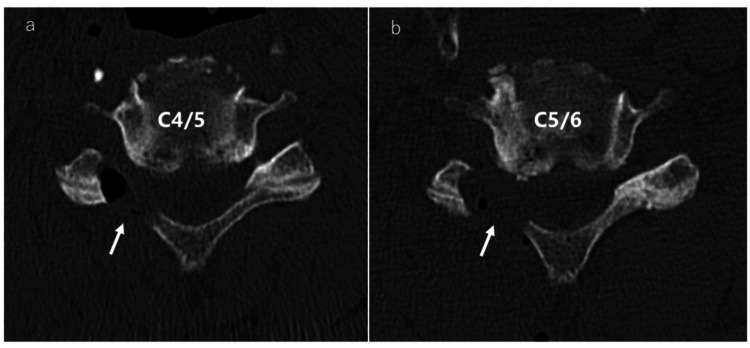
Postoperative CT axial images Posterior decompression is achieved beyond the Luschka joint. The arrows show the decompression sites a: Postoperative CT axial image at C4/5. b: Postoperative CT axial image at C5/6 CT: computed tomography

Subsequently, the superior articular process was cut approximately 7-10 mm outward from the medial edge to achieve decompression beyond the Luschka joint (also known as the uncovertebral joint) (Figures 7-11) using a long-curved high-speed drill and curved Kerrison rongeurs. The extent of bone resection should be measured using preoperative CT. The cranial and caudal pedicles are considered the endpoints of cranial-caudal decompression of the nerve root. When significant bleeding from the internal venous plexus was anticipated after posterior bony decompression, we avoided the removal of perineural membranes. After ensuring that the nerve root was adequately decompressed, hemostasis and irrigation were completed, a drain was placed, and the fascia and the skin were closed in layers with 2-0 Vicryl (Johnson & Johnson, New Brunswick, NJ) and Steristrips (3M Company, Maplewood, MN).

**Figure 12:**
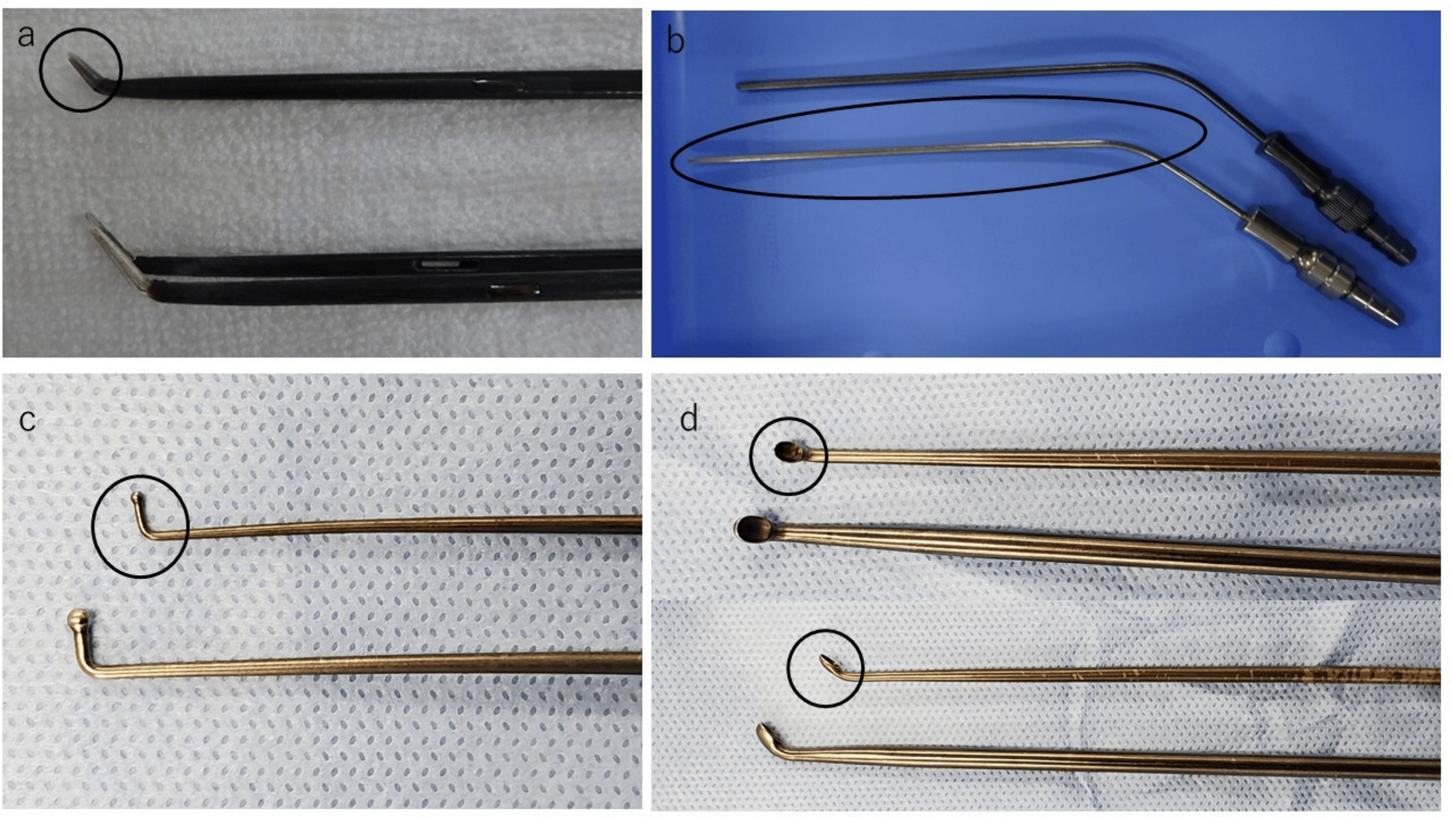
Details of the Fine Instrument (Tanaka Medical, Tokyo, Japan) a: Curved bipolar coagulator. b: Long suction instrument. c: Ball-tipped probe. d: Curette

We used a Fine Instrument (Tanaka Medical, Tokyo, Japan; Figure 12) that employed a 12-mm tubular retractor designed to enable fine procedures within a limited working space.

## Discussion

This is the first report in the literature to describe applying the 12-mm tubular retractor of SYNCHA microendoscopic system for the treatment of CSR. We conducted the surgical procedure for seven cases (nine levels) of CSR between January 2022 and December 2023. Patient demographics and clinical outcomes are presented in Table 1. The follow-up period was at least six months. Clinical records were retrospectively analyzed. We evaluated the following parameters: (1) the numerical rating scale (NRS) for neck and upper limb pain before surgery and at the final follow-up; (2) operating time per single level; (3) intraoperative blood loss per single level; and (4) perioperative complications.

**Table 1:** Patient demographics and clinical outcomes CSR: cervical spondylotic radiculopathy; NRS: numerical rating scale

Disease	No.	Age, years	Sex	Level	Follow-up period (months)	Preop. NRS of neck pain	Postop. NRS of neck pain	Preop. NRS of arm pain	Postop. NRS of arm pain	Operating time (mins)	Blood loss (mL)
CSR	1	46	M	C5/6	12	4	1	8	1	90	16
	2	56	M	C4/5	6	5	2	7	0	84	14
	3	51	M	C5/6	12	2	0	8	0	78	10
	4	59	F	C5/6	12	6	2	8	1	81	16
	5	41	M	C5/6/7	6	5	1	5	0	132	12
	6	44	F	C4/5	6	6	0	9	0	84	3
	7	65	M	C4/5/6	6	5	0	8	0	158	21

**Figure 13:**
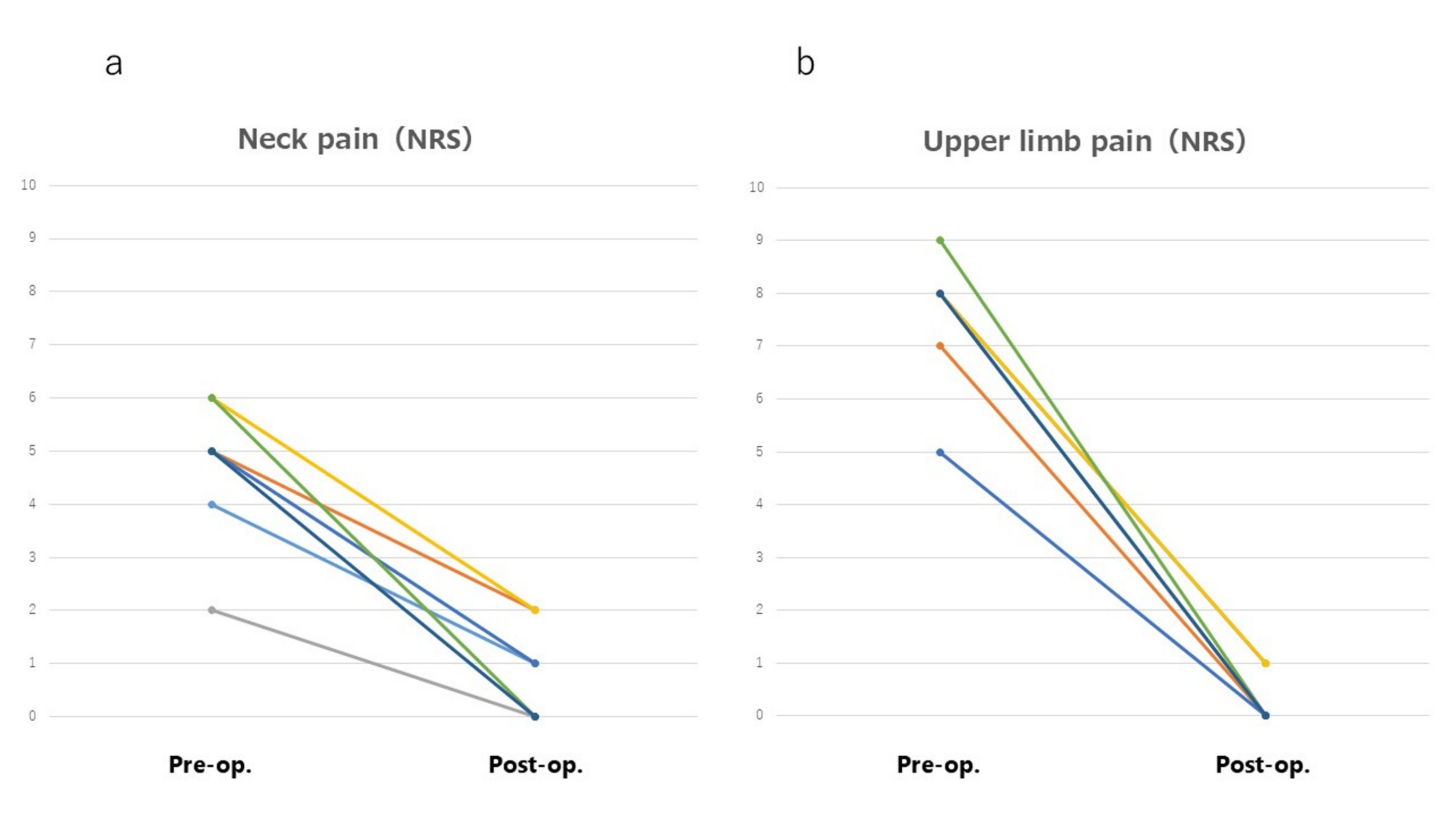
NRS scores for neck and upper limb pain The scores were found to be improved at the final follow-up in all CSR cases a: NRS scores for neck pain. b: NRS scores for upper limb pain CSR: cervical spondylotic radiculopathy; NRS: numerical rating scale

The NRS scores for neck and upper limb pain were found to be improved at the final follow-up in all CSR cases (Table 1, Figure 13). The mean NRS scores for neck pain were 4.7 and 0.9 points before surgery and at the final follow-up, respectively (Figure 13a). The mean NRS scores for upper limb pain were 7.5 and 0.3 points before surgery and at the final follow-up, respectively (Figure 13b). The mean operating time and intraoperative blood loss per single level were 78.6 minutes and 10.2 mL, respectively (Table 1). No dural tear or CSF leakage occurred in any of the cases. No other perioperative complications including neurological deterioration were observed.

We have previously reported the usefulness and minimal invasiveness of decompression surgery using the METRx microendoscopic system with the 16-mm tubular retractor [2,4]. In this study, we used the 12-mm tubular retractor of the SYNCHA microendoscopic system for more minimal invasiveness instead of the 16-mm tubular retractor to perform posterior decompression surgery for the treatment of CSR. The SYNCHA novel microendoscopic system was developed by Yoshida in 2020. Thanks to its joystick action, the 12-mm tubular retractor provided a consistently equivalent field of view compared to the 16-mm tubular retractor. Despite the limited working space, it was possible to perform adequate decompression by using the 12-mm tubular retractor. Although decompression surgery using a 12-mm tubular retractor appeared to take longer operating time compared with the 16-mm tubular retractor, we considered surgeries using a 12-mm tubular retractor to be more minimally invasive techniques because these could be performed with a smaller skin incision and the intraoperative blood loss was comparable to a 16-mm tubular retractor. Furthermore, satisfactory clinical outcomes were obtained while employing a 12-mm tubular retractor.

Additionally, we highlight that using the 12-mm tubular retractor has the following advantages over FESS [5-7] and BESS [8-10]: (1) The 12-mm tubular retractor of the SYNCHA microendoscopic system is fixed to the table-side rail, allowing the use of both hands, whereas in FESS and BESS, one hand must hold a microendoscope, and decompression procedures, including dissection of the fibrous adhesions around the dura mater and nerve roots, can only be performed with the other.

(2) The 12-mm tubular retractor of the SYNCHA microendoscopic system does not require continuous irrigation with normal saline, and hence there is no concern regarding elevated cerebrospinal fluid pressure.

(3) The SYNCHA microendoscopic system can be applied to cervical and thoracic myelopathy.

(4) The SYNCHA microendoscopic system can be also applied through the transforaminal approach at the L5/S level, whereas in FESS, the iliac bone interferes, making it difficult to insert the microendoscope.

(5) Minor dural tears can be easily detected and repaired using the SYNCHA microendoscopic system.

(6) The ability to use both hands allows the surgeon to secure the surgical field while suctioning blood and using bipolar forceps, and manage sudden massive bleeding from the venous plexus in the 12-mm tubular retractor of the SYNCHA microendoscopic system.

(7) Because irrigation with normal saline is not required in the 12-mm tubular retractor of the SYNCHA microendoscopic system, placement of hemostatic agents, such as topical gelatin-thrombin matrix sealant, is possible.

(8) The SYNCHA microendoscopic system allows multilevel spinal surgeries and tandem operations.

When using a 12-mm tubular retractor, we encountered no perioperative complications such as a dural tear, CSF leakage, or neurological deterioration. In case of an intraoperative dural tear, it is possible to perform dural suturing microendoscopically through a 16-mm tubular retractor. However, it is currently unclear whether dural suturing can be performed through a 12-mm tubular retractor. We believe that the patch technique alone, with Vicryl mesh (Ethicon, Raritan, NJ) and fibrin glue [11] may be possible within a 12-mm tubular retractor.

The limitations of this technical note are as follows: (1) the small sample size; (2) the short follow-up period; (3) the outcome measure is limited to neck and upper limb pain, with no use of other standard scores; and (4) there is no direct comparison with the 16-mm tubular retractor, FESS, and BESS.

## Conclusions

We are currently treating CSR cases by performing posterior decompression surgery using a 12-mm tubular retractor of the SYNCHA novel microendoscopic system. Although this study had a small sample size as it is just a technical note, we believe that our preliminary data show that the microendoscope-assisted posterior decompression technique using the SYNCHA microendoscopic system with a 12-mm tubular retractor is not only safe, minimally invasive, and effective for the treatment of CSR, but it may also provide advantages over FESS and BESS. Future studies with larger sample sizes, long-term follow-up, and randomized prospective comparison with the 16-mm tubular retractor, FESS, and BESS are necessary to confirm the advantages of this technique.
